# Social Network Analysis and Resilience in University Students: An Approach from Cohesiveness

**DOI:** 10.3390/ijerph15102119

**Published:** 2018-09-26

**Authors:** Cristina Liébana-Presa, Elena Andina-Díaz, María-Mercedes Reguera-García, Iván Fulgueiras-Carril, David Bermejo-Martínez, Elena Fernández-Martínez

**Affiliations:** 1 SALBIS Research Group, Faculty of Health Sciences, Nursing and Physiotherapy Department, Universidad de León, 24401 León, Spain; cliep@unileon.es (C.L.-P.); mercedes.reguera@unileon.es (M.-M.R.-G.); ivafulcar95@gmail.com (I.F.-C.); dbermmar01@gmail.com (D.B.-M.); elena.fernandez@unileon.es (E.F.-M.); 2 SALBIS Research Group, Nursing and Physiotherapy Department, Universidad de León, Campus Vegazana S/N, 24071 León, Spain

**Keywords:** nursing students, resilience, social network analysis, cohesion

## Abstract

The Social Network Analysis offers a view of social phenomena based on interactions. The aim of this study is to compare social reality through the cohesion variable and analyse its relationship with the resilience of university students. This information is useful to work with the students academically and to optimise the properties of the network that have an influence in academic performance. This is a descriptive transversal study with 90 students from the first and third year of the Nursing Degree. Cohesion variables from the support and friendship networks and the level of resilience were gathered. The UCINET programme was used for network analysis and the SPSS programme for statistical analysis. The students’ friendship and support networks show high intra-classroom cohesion although there are no differences between the support networks and friendship or minimal contact networks in both of the courses used for the study. The network cohesion indicators show less cohesion in the third year. No correlations were found between cohesion and resilience. Resilience does not appear to be an attribute related to cohesion or vice versa.

## 1. Introduction

Human beings interact daily with a vast network of people, and the relationships that we establish among ourselves play an important part in our lives. Regarding this, we can find studies that prove that having strong relationships is essential for our health and well-being [1]; that the diversity in social interactions appears to correlate with better health [2]; that we can experiment with more positive affection when we feel better connected with others [3]; or even that the quantity and the quality of our social relationships can reduce the risk of mortality [4]. 

Due to the complexity of social reality and the exhaustive search of the comprehension of social phenomena, we must carry out different approaches and analysis strategies [5]. The theoretical and methodological paradigm of the Social Network Analysis (SNA) allows us to (a) evaluate the context of the relationships in an empirical manner, and (b) record contexts of social interactions that (c) determine the behaviour of the people that are a part of said contexts [6]. 

Within this methodological and research context, the analysis of interactions can be carried out from a group perspective. That is, studying the intensity of interactions within a group, the level of consensus, or the feeling of belonging. All of this is a reference to a component of the SNA, known as cohesion. Cohesion is the combination of connections and relationships among individuals, groups, and organisations within a community, and it implies interdependence and shared commitments among said members [7]. The idea of cohesion for Borgatti, Everett and Johnson is “connectedness or ‘Knittedness’. Perhaps the simplest measure of cohesion is density. Density is simply the number of ties in the networks expressed as a proportion of the possible number. Some researches prefer to use the average degree of the networks. In this context, the level of ‘cohesion’ depends on, for example, frequency, affinity (homophily), geodesic distance (or proximity), and so on” [8] (pp. 150–151).

The in-depth analysis of relationships or networks, from a cohesive point of view, can be interesting in young people, due to the influence that the group of friends has during this stage, and on their lifestyles [9]. Specifically, in university students, the start of a new stage in their studies means a transformation of their educational and personal context [8]. Knowing and identifying with groups of equals will make the experiences and feelings of belonging with these groups much more pleasant [10], causing them to partially move away from their familiar group [11]. Therefore, having knowledge of the cohesion in their group of equals, both inside and outside of the classroom, can help to identify individuals at risk of exclusion, of suffering from bullying, or at risk of acquiring non-healthy lifestyles [11]. For the teachers, this knowledge can help them to build structural intervention strategies within the classroom [12], as well as personalised educational interventions, thus helping with the integration of isolated students [13].

We have several metrics that are focused on studying the cohesion of a group. These tend to be used to establish comparisons between networks of different kinds and between the same networks in multiple time frames using transversal analysis, lineal analysis, or both [14]. 

One of these is homophily, known as the tendency that individuals have to relate to other, similar individuals [15]. We discovered some studies that show that homophilic friends have closer, much more satisfactory and stable relationships throughout their lives, compared to those who are not homophilic [16]. This is an essential characteristic, not only in friendship networks but also in networks that exchange help and support. The more homophily there is in a group, the better the attitudes and positive feelings of belonging. This favours the resolution of disruptive disagreements, the achievement of consensus, and it promotes a positive view of the group as a social unit [17]. Loomis’ classic study [18] reflects that homophily relates to psychological characteristics such as intelligence, attitudes, and aspirations. People who have homophilic relationships share common characteristics such as beliefs, values, education, etc. which makes communication and the creation of a relationship much easier. On the other hand, homophily also allows a person to know what attributes are seen as important within a group [18]. Similarities encourage people who are a part of a group in which friendship is formed, favouring the acceptance of pairs [19]. All these statements are highly interesting in communities of young university students. 

Another metric to consider in cohesion is the number of relationships that an individual has in their personal network in relation to all of the possible relationships [20,21], which is known as the ego density. So, we found some studies which point out that extroverted people tend to stay linked to their friends in their network, as well as promoting meetings between different people [22]. This fact is important because, even though it is a term related to the individual, it has been used in different studies to be able to explain group behaviour. Each individual is part of the behaviour in a group, in the same sense that group behaviour defines each individual [8]. 

The cohesion of a group interferes with motivation. The most united members of a group are more motivated to stay, participate and contribute to the well-being of the group [23]. On the other hand, the cohesion in a group affects the confrontation and the resistance of the members to this, and these abilities are linked to resilience. Resilience is defined (a) as an ability to live and evolve positively, in a socially acceptable way, despite the stress caused by an adversity, or (b) as an interaction process of an individual with his surroundings, which concludes with the individual adapting to this [24].

We could not find a lot of publications that address group aspects and their implications of resilience [25,26,27]. In the subject that concerns us, the group of university students, one of the most recent studies states that the position that a university student holds in their contact network shows a positive correlation with resilience [28]. This could mean that individual resilience could be determined by the position that the person holds within a network. The results of these investigations can be taken into consideration by managers and designers of public politics in educational material, with the aim of creating positive environments that facilitate the integral development of the student body [12]. The study of resilience in university students has special significance in students studying for a career in health sciences. These young people feel high levels of stress, given that they have to adapt to the experiences they go through during their clinical practices, such as the chronic illness of a patient or even the death of one. This stress can increase when, as well as putting the theoretical knowledge that they have acquired into practice, they also have to meet the needs and demands of their patients [29]. Due to this, resilience could be an essential skill to succeed in their clinical experience and face their future labour [30]. The existence of correlation between resilience and cohesion may make it possible to ascertain what contact patterns are aligned with resilience. Such insight may be applied to educational methodologies, not only to improve academic performance, but also to create emotionally sustainable networks. 

Because of this, it is interesting to carry out studies in which variables such as resilience are linked to the analysis of cohesion within a group, specifically in health sciences students. In virtue of the aforementioned, this study has the following aims:

– To compare the intra-classroom cohesion in friendship and support networks belonging to students in the first and third years of a Nursing Degree.
– To analyse the relationship between the cohesion and resilience variables of university students.

## 2. Materials and Methods 

This investigation is a descriptive, transversal study. 

### 2.1. Description of the Sample

The number of students from a Nursing Degree from a Spanish Public University that participated was 90. The characteristics of the study can be seen in Table 1. The study involved the participation of 48 students from the first year and 42 students from the third year. The first and third years were chosen because it is intended to determine the differences between a new network, formed by nodes that have been known for a short time, and a more mature one, with several years of relationship. Men comprised 21.1% of the students and 78.8% were women. 

### 2.2. Instruments Used to Collect Data and Variables

Data collection was carried out through a questionnaire that was designed *ad-hoc* and filled in by individuals, in which social-demographic data was collected, such as the gender and the academic year of the student. 

The cohesion variable was described using the following metrics: density, reciprocity, average distance, diameter, homophily, ego density and accessibility of each one of the course years (Table 2). The networks in which the cohesion was analysed were friendship networks [31] and support networks [32]. To calculate the cohesion, a limited census of actors was used and the replies were carried out on a list of students from each course year. The replies were evaluated on a Likert scale from 0 to 4 [28], with the support network being 0 for “never” and 4 for “always”. On the friendship network, 0 corresponded to “no friends” and 4 to “best friend”. 

The resilience variable was measured with the abbreviated version of Campbell-Sills and Stein (2007), validated in Spanish young people [35], from the original Connor-Davidson scale [36]. The questionnaire had 10 items. The results obtained were quantified with the Likert scale that went from 0 to 4 points, with regards to the level of agreement or disagreement, respectively. 

### 2.3. Procedure

The data used for this study was obtained during the first semester of the 2016–2017 academic year. 

To examine the cohesion variable of the social-centric networks of the classroom, we needed to elaborate on the charts from the first and third year separately. In these charts, both friendship and support relationships are shown. Square charts were designed with N rows × N columns, where the data was placed. To calculate the cohesion, we had to introduce dichotomised data. In the support network, we opted for the following dichotomisation criteria: the results 0 meaning “never” and 1 meaning “rarely”, that which corresponds to “does not ask for help” would be represented with 0, whilst the values 2, meaning “sometimes”, 3, meaning “frequently”, and 4, meaning “always”, were represented with 1, which corresponds with “does ask for help” [28,37]. 

With regards to the intensity, in the case of the Friendship Network, the data coding linked two different networks [28,38]:

In the “Minimal Contact Network” the points from 1, “hardly any”, 2, “some”, 3, “a lot” and 4, “good friends” were dichotomized with 1.

– Meanwhile, in the network of “Friendship Network” the concept was restricted, just values 3 and 4 were considered as 1.

### 2.4. Data Analysis

The charts obtained with the data collection were carried out with an Excel programme. To calculate the cohesion dimensions (density, reciprocity, average distance, diameter, ego density, homophily, and accessibility), the UCINET V. 6.645, Analytic Technologies, Lexington, KY, USA, a software tool for the analysis and visualization of exploratory data networks [39], was used. Once the cohesion data was obtained, the SPSS program (V.24, IBM, New York, NY, USA) was used to carry out the statistical analysis. The descriptive statistics were calculated, as were the Pearson correlations between the cohesion variables (ego density) and resilience. In addition, the data obtained in the networks were checked through the Mann-Whitney U Test. 

### 2.5. Ethical Considerations

The participants were verbally informed of the aims of the study and, to be able to participate in the study, they were required to voluntarily sign an informed consent document. The confidentiality and anonymity of the subjects that were a part of the study were taken into account at all times. The Deontological Code and the Helsinki Declaration were followed, as well as the legal regulations of data confidentiality (Law 15/1999 of the 13th of December on data protection of personal character). This study was approved by the Ethical Committee of the University (ETICA-ULE-010-2017), which guaranteed the compliance of all ethical and legal matters. 

## 3. Results

Below, the cohesion indicators normalised of support and friendship networks from the first and third years are detailed in Table 3. 

The results obtained for the cohesion indicators of support networks and friendship and minimal contact networks in the first and third years, show less cohesion in the higher year. 

The average obtained by the resilience variable in the first course was of 29.42 and the standard deviation was 5.18. With regards to the third year, the results were 28.98 and 5.18 respectively.

**Figure 1:**
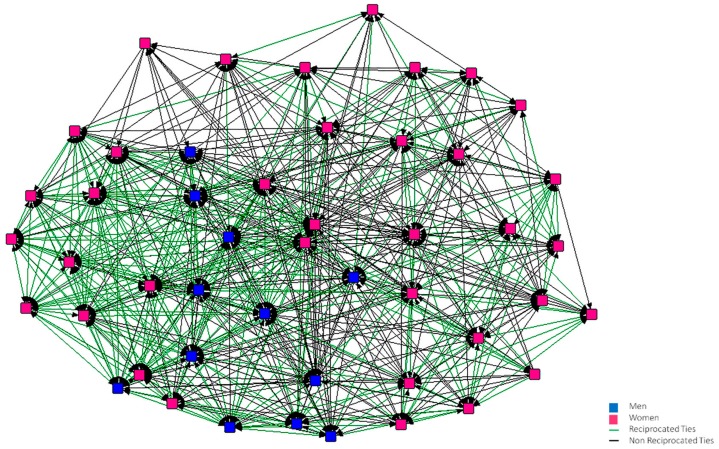
Graph of the first year Support Network.

**Figure 2:**
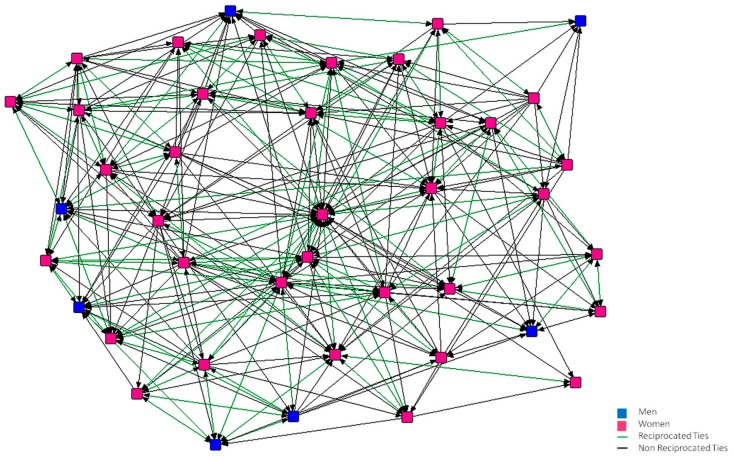
Graph of the third year Support Network.

**Figure 3:**
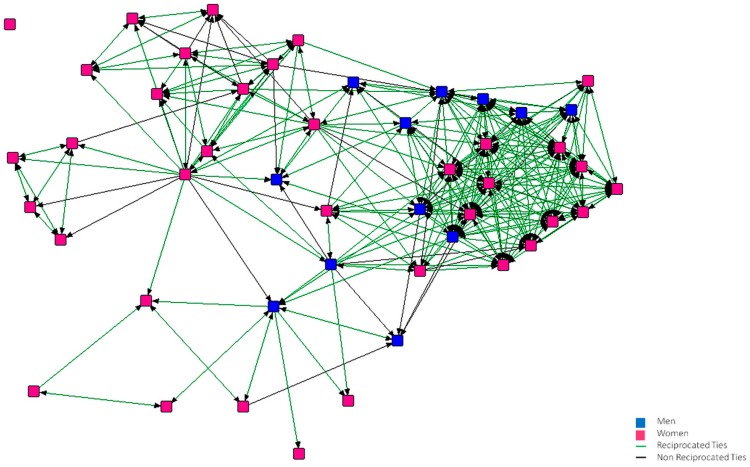
Graph of the first year Friendship Network.

**Figure 4:**
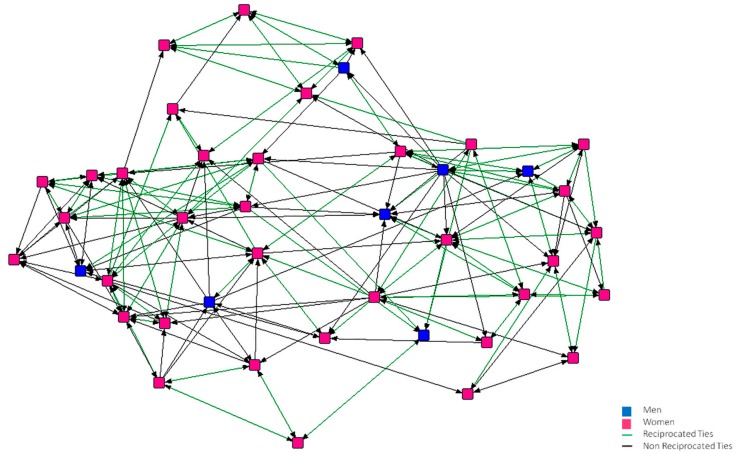
Graph of the third year Friendship Network.

**Figure 5:**
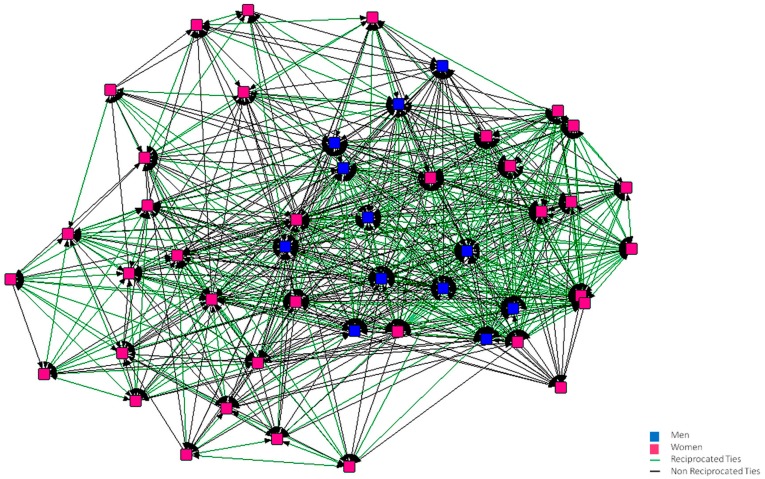
Graph of the first year Minimal Contact Network.

**Figure 6:**
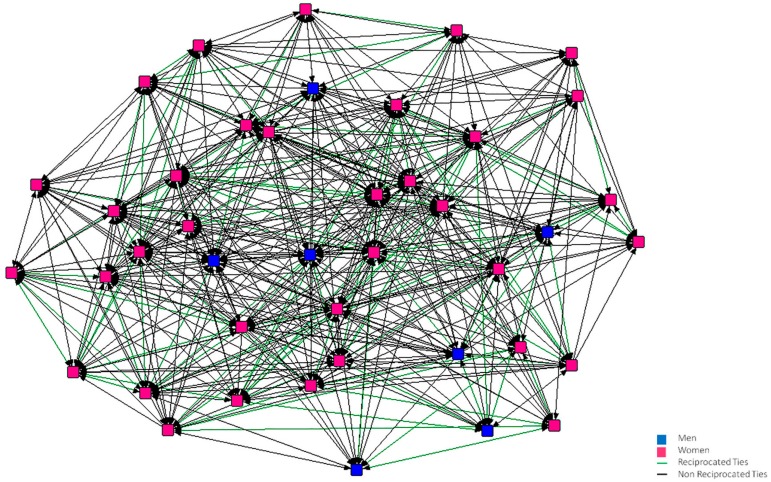
Graph of the third year Minimal Contact Network.

In Figure 1, Figure 2, Figure 3, Figure 4, Figure 5 and Figure 6, we can see the graphic representations of the support and friendship networks of the first and third years, with the non-mutual relationships being shown in black and the mutual relationships in green. Men are represented in blue and women are represented in pink. 

With regards to the accessibility of the support network in both years, as can be seen in Figure 1 and Figure 2, all the nodes are required by other nodes and all of them turn to at least one person in the case that this person needed help. 

In the friendship network of the first year (Figure 3), there are three nodes that did not consider anybody to be good friends and one of them is also not considered by anybody as a good friend, thus becoming isolated. In the third year network (Figure 4), two nodes do not consider themselves as friends of anybody, although there are other nodes that consider these to be good friends. 

With regards to the accessibility, in the minimal contact network of the first year (Figure 5), one node considered nobody as a friend, although he was considered as a friend by other students. In the third year network (Figure 6), all of the nodes were accessible and helped other students. 

Upon comparing the networks of both years through the Mann-Whitney U Test, the results were that there are no significant statistical differences between the cohesion variables of the support, friendship and minimal contact networks (*p* = 0.317). 

Below, the correlation analysis between individual scores of the density of each node (ego density) is shown in detail, as well as the resilience in each one of the friendship and support networks from both groups. This correlational result does not confirm a lack of relationship, but confirms that there was no statistical relationship between cohesion and resilience in this study (Table 4). 

## 4. Discussion

This study describes and compares the cohesion and resilience variables between two years of the university students studying a Nursing Degree. 

The results obtained show a high level of cohesion that exists in both years in the support network. In the friendship network, we can see that the cohesion is high but lower than that in the minimal contact network, as is logical given that it is a network that is characterized for having fewer friends, but with stronger relationships. 

The density of the support network from the first year is very close to the possible maximum (0.980) and shows a slight decrease in the higher year (0.799), as was expected given that the third year has more acquired professional skills. Ramos-Vidal pointed out that the high density of the cohesion of each student provides the student with a sense of security and support [15], which could be the situation of the first year in which they stated that they felt insecure in the new academic university environment that they had to face. In this sense, the study carried out by Requena, indicated that the density level in friendship networks in the classroom was directly proportional to the number of positive feelings with regards to the class as a whole and, therefore, it is an indicator of the classroom cohesion [40]. This situation should be especially useful if the density was high, as we can see in our study. 

The minimal contact networks showed high density (in the first and third years 0.5 and 0.42 respectively). The friendship networks showed lower values (the first year 0.17 and the third year 0.12), than expected. This means that each node received the friendship nomination from a considerable amount of classmates. Our data support that the social organisation in the microsystems in the field of education reinforce the sense of community increasing the sense of rooting and interdependence [41]. However, the high density could also be associated with a feeling of restriction, loss of autonomy and the feeling of being trapped. This could be due to the fact that their friends are all together in a friendship group, and they can come across difficulties if they are in disagreement with a friend. 

With regards to the reciprocity of the support network, as was to be expected in a network with an elevated density, there was a high percentage of relationship strength. In the case of asking for help, there was not a single node that would not ask a companion for help, nor were there any nodes that would not help another companion. This could mean that, although the information that exists within a class can be accessed by all, the students relate to each other to compare the interpretation of that information and perform the learning based on group problems and, therefore, there are no isolated nodes, improving connectivity and the sense of community when working in small groups within the classroom as it happens among university students of Medicine [42].

To analyse the reciprocity of the minimal contact network, sub-groups were examined through dyads [33]. On the one hand, a high number of dyads can mean a high level of cohesion, given that they indicate the several mutual relationships exist. But, on the other hand, if said mutual dyads are only established among individuals, there would not be a high level of cohesion. To be able to determine if the reciprocity is a reliable indicator of cohesion, we also analysed the isolated nodes through the accessibility that were observed in the network figures. The reciprocity was higher in the minimal contact network in both years, with higher consistency being discovered between the nodes who considered each other to be mutual friends. This, in part, could be because there was high density and almost all of the students considered the rest as not really friends. 

The reciprocity in the friendship network was high, with the third year being the lowest in score, contrary to the minimal contact network. This implies that, in the third year, the students who consider ten companions to be very good friends, the same level of relationship is only mutual with three other companions. Additionally, it is characterised because it is the network that reached the longest distances in average distance and diameter. 

The diameter of the support network of both years is 3 and the average distance of the first year was smaller than that of the third year (1.6 and 1.7 respectively), which indicates a high level of cohesion in both groups. Although the average distance continued to be slightly higher, we can confirm that the level of cohesion was still high as well. 

The diameter and the distance in the minimal contact network in year three were high, which shows that, over time, there can be more relationships among individuals and that these can be of a more positive nature [43]. 

The heterophily was high in all of the networks; there is no tendency of people associating themselves with other, similar people. This fact, according to authors such as Scatà and collaborators [44] can prove that said tendency plays a double role in contraction, with this being reinforcing or delaying the noxious ideas that can lead to unhealthy behaviours such as suicide. In addition, these values are justified because the majority of the students belong to different geographic areas [45]. 

With regards to the resilience variable we found descriptive values similar to those of the study carried out by Fernández-Martínez and collaborators [28] and slightly lower in the study carried out by Rios and collaborators [46] in the university body. Resilience in college students is intimidated by success. The determinants of success are self-control (the ability to regulate attention, emotion and behavior in the presence of temptation) and grit (the tenacious pursuit of a self-imposed goal despite setbacks) [47]. Therefore, these skills must be rigorously analyzed in the university community. 

The accessibility was high in all of the analysed social networks due to the scarce amount of isolated nodes detected; this reinforces Friendkin and collaborators’ ideas that state that, if a high amount of isolated people exist, there will not be any cohesion [12]. Only one node existed in the friendship network of the first year, who did not consider anybody to be a friend, but who is considered to be a friend by other companions. Although we did not see the same behaviour in the minimal contact network, it is possible that this person is an introvert, a person who does not have the same concept of friendship as the rest of the students or does not have any incentive or reason to mingle with the rest of the group [34]. 

Very few isolated nodes were discovered in all of the networks that were analysed for this study, so the dyads established in the set of networks and the reciprocity were reliable enough to be able to determine the network cohesion [33]. 

We did not find significant correlations between resilience and cohesion in the different networks. This indicates that, in both years, the relationships established are highly cohesive, but that one student who belongs to a group of friends, who mutually look after and care for each other, does not contribute to its resilience. We have not observed that the most resilient people have higher ego density within both friendship and support networks. On the contrary, in other groups, for example, the army, the resilience shows a positive correlation with network cohesion [25]. The high cohesion values hint that a high amount of homophily with some attribute will exist [10]. On the other hand, the low level of homophily correlation with regards to resilience shows that this skill is not considered as an important one among nursing students to be able to establish friendships or to ask for help [9]. 

This investigation shows limitations that indicate that the results should be interpreted with caution. The metrics that measure the network cohesion are many and not all of them have been analysed in this study. In addition, the network cohesion data in the nursing degree cannot be generalised for its use with the rest of the university students. We have not carried out analysis relative to the gender differences of the cohesion data, as has been done in other network investigations [48]. 

For future lines of investigation, we propose to continue relating resilience with other cohesion variables, including the study of subgroups and roles, and individual cohesion variables, such as the diameter or the average distance. On the other hand, given that from the comparison between the networks of the first and third years, we can discover differences and similarities, we could consider carrying out a longitudinal study to determine the temporal evolution of the network structure. With said study, we could assess classroom dynamics and how these adapt to the respective years. Likewise, we would measure the evolution of resilience, being able to connect this with network evolution. A complete description of the network will provide us with all of the necessary information to be able to take measures that are not only educational but measures of any kind adapted to the specific class. Lastly, it would be interesting to be able to compare our data with other university degrees. 

## 5. Conclusions

We have managed to obtain a result for the aims suggested in this study: to compare the intra-classroom cohesion in the friendship and support networks of students in the first and third years of the nursing degree; as well as analysing the relationship between cohesion variables and resilience in university students. 

We did not find statistically significant relationships between resilience and cohesion in the university students. For this group, the resilience is not an attribute that is affected by cohesion. 

The friendship and support networks of the students show high intra-classroom cohesion, although there are no differences between support networks and friendship and minimal contact networks in both of the years presented in this study. These high cohesion values suggest that a high level of homophily with some attributes will exist. This fact is interesting enough to be studied more in depth, with the aim of boosting group values and working with them. On the other hand, the high cohesion provides students with interesting social capital that can make the creation of ideas and resources easier, as well as contributing to their academic performance inside and outside the classroom. 

## Figures and Tables

**Table 1:** Student characteristics.

Course	**Year**	**Gender**		**Total (%)**
		**Male**	**Female**	
	First year	12	36	48 (53.3%)
	Third year	7	35	42 (46.6%)
	Total (%)	19 (21.1%)	71 (78.8%)	*N* = 90 (100%)

**Table 2:** Cohesion metrics and conceptual definitions.

Metrics	Conceptual Definition
Density	Proportion of possible relationships that are in the network. The density values vary between 0 and 1, with 1 being when all of the possible relationships are present [9,33].
Reciprocity	This measures the strength of the relationship and the percentage of all the mutual links in the network [9,33].
Average distance	Average point of all the distances in a network [33].
Diameter	This is the length of the largest distance between two nodes in a network [9,33].
Ego Density	This is the number of relationships that an ego has in their personal network, in relation to all the possible ones [23,34].
Homophily	Homophily is the tendency that individuals have to associate and link themselves with similar people [3]. The E-i index, which is used to measure homophily, has values between −1, which means that the network is homophilic, and +1, which reflects a heterothallic tendency.
Accessibility	Nodes are accessible if a path exists between them. To define accessibility, we have to consider the different paths. Specifically, if a path goes from A to B, then B is accessible from A [9].

**Table 3:** Descriptive indicators of the cohesion in networks from the first and third years.

Network	Course	Cohesion				
		Density	Reciprocity	Diameter	Average Distance	Homophily
Support Network	First year	0.980	0.55	3	1.6	0.895
	Third year	0.799	0.49	3	1.7	0.904
Minimal Contact Network	First year	0.5	0.55	3	1.6	0.881
	Third year	0.42	0.42	4	2.1	0.921
Friendship Network	First year	0.17	0.48	9	3	0.914
	Third year	0.12	0.35	12	3.5	0.898

**Table 4:** Correlations between resilience and ego density in the support and friendship networks in the first and third years.

Resilience/Year	Correlations	Minimal Contact Network	Friendship Network	Support Network
Resilience First year	Correlation of Pearson	0.036	0.014	0.114
	Significant(bilateral)	0.809	0.922	0.441
Resilience Third year	Correlation of Pearson	−0.58	−0.142	−0.231
	Significant(bilateral)	0.714	0.364	0.135
